# Screening and Phenotyping of Lactic Acid Bacteria in Boza

**DOI:** 10.3390/microorganisms13081767

**Published:** 2025-07-29

**Authors:** Xudong Zhao, Longying Pei, Xinqi Wang, Mingming Luo, Sihan Hou, Xingqian Ye, Wei Liu, Yuting Zhou

**Affiliations:** 1 Experimental Teaching Demonstration Center of Food Safety and Nutrition, Xinjiang Institute of Technology, Aksu 843100, China; moreonce0@163.com (X.Z.); peilongy@163.com (L.P.); 19326758914@163.com (M.L.); psu@zju.edu.cn (X.Y.); lw520233831xx@163.com (W.L.); 2 College of Food Science and Engineering, Tarim University, Alar 843300, China; 3 Xinjiang Auricularia Engineering Technology Research Center, Aksu 843100, China; 4 Shaanxi Grain and Oil Research Institute, Xi’an 710082, China

**Keywords:** Boza, lactic acid bacteria, isolation, identification, fermentation properties, probiotic properties

## Abstract

The aim of this study was to isolate and identify lactic acid bacteria (LAB) from a traditional fermented beverage, Boza, and to conduct an in-depth study on their fermentation and probiotic properties. The fermentation (acid production rate, acid tolerance, salt tolerance, amino acid decarboxylase activity) and probiotic properties (gastrointestinal tolerance, bile salt tolerance, hydrophobicity, self-aggregation, drug resistance, bacteriostatic properties) of the 16 isolated LAB were systematically analyzed by morphological, physiological, and biochemical tests and 16S rDNA molecular biology. This analysis utilized principal component analysis (PCA) to comprehensively evaluate the biological properties of the strains. The identified LAB included *Limosilactobacillus fermentum* (9 strains), *Levilactobacillus brevis* (2 strains), *Lacticaseibacillus paracasei* (2 strains), and *Lactobacillus helveticus* (3 strains). These strains showed strong environmental adaptation at different pH (3.5) and temperature (45 °C), with different gastrointestinal colonization, tolerance, and antioxidant properties. All the strains did not show hemolytic activity and were inhibitory to *Staphylococcus aureus*, and showed resistance to kanamycin, gentamicin, vancomycin, and streptomycin. Based on the integrated scoring of biological properties by principal component analysis, *Limosilactobacillus fermentum* S4 and S6 and *Levilactobacillus brevis* S5 had excellent fermentation properties and tolerance and could be used as potential functional microbial resources.

## 1. Introduction

Boza, an intangible cultural heritage of the Kirghiz people in the Xinjiang province of China, is a traditional fermented beverage prepared through a natural fermentation process using grains such as barley, corn, millet, or oats [1]. Boza is rich in a variety of bioactive substances with health benefits, including antioxidant, anti-inflammatory, and immune system boosting properties [2], and is widely popular among international vegans and lactose-intolerant people. Cereal ingredients are rich in such nutrients, such as amino acids, proteins, vitamins, minerals, and dietary fiber, which provide an ideal incubation environment for the growth of microorganisms [3]. The addition of LAB enhances the functional properties of cereals, and their growth and metabolism during fermentation not only promoting food digestion and absorption but also enhancing the bioavailability of micronutrients such as iron, zinc and calcium. Furthermore, LAB inhibits the growth of pathogenic bacteria [4], synthesizes essential short-chain fatty acids and other low molecular organic components [5], and releases phenolic compounds to increase the antioxidant activity of fermented foods [6], which has a significant impact on the quality changes in fermented cereals.

LAB are abundant in naturally fermented Boza and play an important role in the fermentation process [7]. To improve and to stabilize the quality of Boza, screening of good indigenous strains as fermenters for inoculation and fermentation is an effective measure to ensure the stable quality of Boza. Ucak et al. [8] found that the abundance of *Enterococcus* spp., *Lactococcus* spp., *Shigella* spp., *Escherichia* spp., *Bacillus* spp., and *Lactobacillus* spp. was high in Boza beverages processed by pre-enrichment. Queiroz et al. [9] screened *Pediococcus pentosaceus* and *Pediococcus acidilactici* from Boza samples in Bulgaria. Andrea et al. [10] isolated *Lactobacillus paracasei* subsp. *paracasei*, *Lactobacillus casei*, and *Lactobacillus parabuchneri* from Sofia Boza by a culturable method. Yigit et al. [11] analyzed the Boza samples in Turkey and found that Lactobacillus plantarum, Pediococcus pentosaceus, and Enterococcus faecium were the dominant bacteria. LAB are absolutely dominant in most naturally fermented cereal beverages. However, there is variability in naturally fermented strains in different geographical areas, which may be related to factors such as environment, temperature, fermentation raw materials, water source, and sample analysis and processing methods in each location [12].

Currently, related research on Boza focuses mainly on its nutritional composition, flavor characteristics, and fermentation process, but less focus is centered on its LAB resources. In the present study, we used the lactic acid bacteria-rich Boza from the Xinjiang region of China as the research target and conducted physiological and biochemical tests and genetic identification of the screened LAB, to determine the fermentation characteristics of the strains, such as the growth and acid production rate, acid resistance, salt tolerance, and amino acid decarboxylase activity, and the beneficial characteristics of the strains, such as gastrointestinal tolerance, strain hydrophobicity and auto-aggregation, antioxidant activity, drug resistance, and bacteriostatic properties. Furthermore, by combining the principal component analysis method, we screened out the candidate strains with excellent comprehensive performance, enriched the strain library, and provided more options for different strains in the preparation of the fermentation agent for the traditional beverage Boza.

## 2. Materials and Methods

### 2.1. Materials and Reagents

Boza was produced in the Urumqi region of Xinjiang, China, and handmade by local herdsmen using traditional fermentation techniques. These two batches of products are completed on the same day according to the same process flow. Three samples were selected from each batch of products and then transported to the microbiology laboratory (Xinjiang Auricularia Engineering Technology Research Center, Xinjiang, China) at 4 °C.

All MRS medium ingredients (MRS broth and agar) and reagents for measuring antioxidant activity (2,2-biazobis(3-ethyl-benzothiazole-6-sulfonic acid) and 2,2-biphenyl-1-picrylhydrazyl) were purchased from Shanghai Yuanye Biotechnology Co., Ltd., Shanghai, China; Bacterial Genomic DNA Extraction Kit (Beijing TIANGEN Biotech Co., Ltd., Beijing, China); Gram Staining Kit (Beijing Solarbio Biotech Co., Ltd., Beijing, China); bromocresol violet (Shanghai Yuanye Biotechnology Co., Ltd., Shanghai, China); All enzymes (pepsin, Trypsin and lysozyme) were purchased from Beijing Solarbio Biotech Co., Ltd., China; the chemicals used for PCR amplification of DNA (2 × EasyTaq PCR SuperMix, Trans2K DNA marker, 6 × DNA Loading Buffer) were provided by Beijing TransGen Biotech Co., Ltd., Beijing, China.

### 2.2. Instruments and Equipment

Model 330-5B Constant Temperature Incubator, Zhejiang Jiulan Scientific Instrument Co., Ltd., Huzhou, China; Model XFS 280C High Pressure Steam Sterilization Kettle, Shaoxing Shangcheng Instrument Manufacturing Co., Shaoxing, Ltd., Shaoxing, China; Model SW-CJ 2FD Ultra-clean Bench, Hangzhou Hongyun Instrument Co., Ltd., Hangzhou, China; Bio-Rad Powerpac Universal SDS-PAGE Electrophoresis Apparatus, Bio-Rad Laboratories, Hercules, CA, USA; Model TG16-WS High Speed Centrifuge. Changzhou Jintan Youlian Instrument Research Institute, Changzhou, China; pHS-3C pH meter, Shanghai Anlin Scientific Instrument Co., Ltd., Shanghai, China; CBM-20A UV spectrophotometer, Shimadzu Corporation, Kyoto, Japan; FA1004 electronic balance, Shanghai Youke Instrumentation Co., Ltd., Shanghai, China.

### 2.3. Isolation and Identification of Lactic Acid Bacteria

To select the appropriate dilution gradient for the separation and identification of lactic acid bacteria, a gradient dilution treatment of the Boza samples was carried out. First of all, the fresh impurities were shaken well, and 25 mL of the sample was taken into a sterile conical flask containing 225 mL of normal saline with a sterile pipette and fully shaken to make a 1:10 sample homogenate. Subsequently, gradient dilution (10^−3^, 10^−4^, 10^−6^) was performed, and 100 μL of samples with appropriate dilution gradients were drawn and coated on an MRS plate containing 0.75% calcium carbonate. Each dilution gradient was made in three parallels and cultured at 37 °C for 48 h under anaerobic conditions. The characteristics of colony color, shape, and smoothness were observed and recorded, and three parallels were made for each dilution gradient and incubated at 37 °C for 48 h. The color, shape, smoothness, and other characteristics of the colonies were observed and recorded. Then, single colonies with different morphology were tested for catalase production, Gram stained, and examined microscopically [13]. Sugar fermentation assa [14] was carried out to validate the initially selected strains. Bacterial suspensions were inoculated at 2% (*w*/*v*) into a medium containing 1% (*w*/*v*) bromocresol violet color and various carbon sources (sorbitol, mannitol, cellobiose, alginate, fructose, glucose, arabinose, lactose, sucrose, and xylose). The incubation was performed at 37 °C for 24~48 h, and the color change of the medium was observed. The utilized substrate changed to yellow, while the unutilized substrate remained purple. The results of catalase negative and accurate sugar fermentation of the single colonies with purple Gram staining were initially determined to be lactic acid bacteria, and the colony shape was observed under a light microscope and preserved in 50% glycerol in a −80 °C refrigerator for backup.

### 2.4. Genetic Identification of Lactic Acid Bacteria

The LAB liquid to be examined was received into MRS liquid medium according to 5% inoculum, anaerobically cultured at 37 °C for more than 12 h, and then activated continuously for 2–3 times for DNA extraction. A bacterial genomic DNA extraction kit was used in this test, and the specific method strictly followed the instructions for use of the kit. 16S rDNA sequence identification: DNA was extracted with the bacterial genomic DNA extraction kit, stored at −20 °C, and the universal primer 27F (5′-GTTTGATCMTGGCTCAG-3′) and 1492R (5′-TACGGYTACCTTGTTACGACT T-3′) were selected for the amplification of bacterial 16S rDNA fragments. PCR reaction system (50 μL): Taq PCR Master Mix 25 μL, template DNA 2 μL, upstream and downstream primers (10 μmol/L) 2 μL, and sterile water 19 μL. PCR reaction conditions: pre-denaturation at 98 °C for 2 min, deconvolution at 98 °C for 10 s, annealing at 55 °C for 30 s, extension at 72 °C for 1 min and 30 s, 30 cycles, the last extension at 72 °C for 10 min, and held at 15 °C. The PCR amplification products were examined by 1% agarose gel electrophoresis. After electrophoresis, observe whether there is a bright band corresponding to the marker. After ensuring successful PCR amplification, the sequencing was performed by Sangon Biological Engineering (Shanghai) Co., Shanghai, China.

MEGA 11 software was used to analyze the sequence data, and sequence homology searches were performed in the NCBI database using BLAST [15]. Species assignment was verified when the sequences showed at least 99% identity with the reference strain sequence.

### 2.5. Fermentation Characterization of Lactic Acid Bacteria

#### 2.5.1. Growth and Acid Production Rates

The acid production rate and growth rate of LAB strains were determined according to the method of Huang et al. [16], with slight modifications. The activated bacterial suspension was added into MRS medium at 2% volume fraction and incubated at 37 °C. The OD 600 nm of the organisms was determined at 0, 4, 8, 12, 16, 20, 24, 36, and 48 h, and the growth curves were plotted. The bacterial suspensions of different fermentation times were centrifuged to obtain cell-free supernatant (CFS). The pH of CFS was determined using a pH meter.

#### 2.5.2. Acid and Alkali Resistance, Salt Resistance, and Temperature Resistance

Resistance analyses of acid resistance, salt resistance, and temperature resistance of LAB were based on the method reported by Liu et al. [17], with slight modifications. The pH of MRS medium was adjusted to 3.5, 4.0, 4.5, 5.0, and 5.5 with dilute hydrochloric acid, and the cultures were inoculated at 2% after autoclaving and incubated at 37 °C for 24 h. The autoclaved MRS medium was accessed to 2% cultures, and incubated for 24 h under the conditions of 4 °C, 20 °C, 28 °C, 37 °C, and 45 °C; 0%, 2%, 4%, 6%, and 8% NaCl were added to the liquid MRS medium, and the cultures were inoculated with 2% after autoclaving and incubated at 37 °C for 24 h. The OD 600 nm value (Ai) of the bacterial solution was determined using the normal culture solution as the control (A). The strain density ratio was used to measure the tolerance ability of the strains in different environments, and the strain density ratio was calculated according to the following formula:

Strain density ratio = (Ai/A) × 100.

### 2.6. Determination of Environmental Tolerance in the Simulated Human Digestive Tract

#### 2.6.1. Bile Salt Resistance

The method of Yang et al. [18] was modified slightly and adjusted appropriately. Specifically, in this experiment, different concentrations (0.1%, 0.2%, 0.3%) of pig bile salt were added to MRS liquid medium, and a control group without pig bile salt was established. All samples were inoculated at 4% inoculation volume and cultured at 37 °C for 24 h. After the cultivation, the optical density of each bacterial solution at 600 nm wavelength was measured. The strain density ratio was used to measure the adaptability of the strain in different environments and calculated according to the formula provided in Section 2.5.2.

#### 2.6.2. Modeling of Gastrointestinal Digestion

The gastrointestinal tract environmental tolerance was referred to the method of Nagpal et al. [19], with slight modifications. Briefly, simulated gastric fluid was prepared by suspending pepsin (3 g/L) in 10 mL of sterile saline (0.85% NaCl, *v*/*v*); pH was adjusted to 2.5. Cultures incubated overnight (≈10^8^ cfu/mL) were inoculated into simulated gastric fluid and incubated for 3 h at 37 °C. Simulated intestinal fluid was prepared by dissolving bile salts (0.3% *v*/*v*) and trypsin (1 mg/mL) in 10 mL of sterile saline (0.85% NaCl, *w*/*v*); pH was adjusted to 8.0. Cultures incubated overnight (≈10^8^ cfu/mL) were inoculated into the simulated intestinal fluid and incubated for 6 h at 37 °C. The number of viable bacteria was measured at 37 °C by continuously diluting the culture and incubating it on MRS agar medium for 24 h.

### 2.7. In Vitro Characterization and Evaluation of Probiotic Properties

#### 2.7.1. Surface Hydrophobicity

The OD 600 nm of LAB suspension was adjusted to 0.6 (OD0) using phosphate buffer solution (PBS), 3 mL of suspension was aspirated, 1 mL of xylene was added as a hydrophobic organic solvent, and the upper aqueous phase was measured by OD 600 nm (OD1) [20] after being placed at room temperature for 10 min and then oscillating and mixing for another 20 min. The formula for the surface hydrophobicity of LAB follows:

(OD0 − OD1)/OD0 × 100.

#### 2.7.2. Surface Acid–Base Charge

Similar to the surface hydrophobicity determination method, the optical density (OD 600 nm) of the LAB suspension was adjusted to 0.6 (OD 0) using phosphate buffered saline (PBS). Then, 3 mL of bacterial suspension was taken, and 1 mL of chloroform was added to determine the acidic charge on the surface of LAB, or 1 mL of ethyl acetate was added to determine the alkaline charge on the surface. The sample was allowed to stand at room temperature for 10 min, and then oscillated and mixed, and continued to stand for 20 min. Finally, the optical density (OD 600 nm) (OD 1) of the upper aqueous phase was determined and calculated according to the formula provided in Section 2.7.1 [21].

#### 2.7.3. Capacity for Self-Aggregation

The LAB suspension OD 600 nm was adjusted to 0.6 (OD0) using PBS, 10 mL of the suspension was aspirated, and OD 600 nm was determined by slowly aspirating 2 mL of the upper layer of the bacterial solution after standing at 37 °C for 2, 4, and 8 h (OD2) [22]. LAB autopolymerization capacity was determined using the following equation:

(OD0 − OD2)/OD0 × 100.

### 2.8. Screening of Lactic Acid Bacteria with Antioxidant Properties

#### 2.8.1. Measurement of DPPH Free Radical Scavenging Capacity

In this procedure, 500 µL of LAB solution was mixed with 500 µL of 0.2 mmol/L DPPH solution (dissolved in anhydrous ethanol), and the reaction was carried out in the dark at 25 °C. After 30 min, the mixture was centrifuged at 8000 r/min for 10 min, and the absorbance of the supernatant was measured at 517 nm (Ad0). For the control (Ad1), anhydrous ethanol replaced DPPH, and for the blank control (Ad2), an equal amount of anhydrous ethanol replaced the sample. Zeroing was carried out with a mixture of equal amounts of distilled water and anhydrous ethanol [23]. Each of the aforementioned groups underwent three parallel experiments. Clearance was calculated as follows:

(1 − (Ad0 − Ad2)/Ad1) × 100.

#### 2.8.2. Measurement of ABTS Free Radical Scavenging Capacity

ABTS and potassium persulfate were dissolved in PBS to prepare 7 mmol/L ABTS solution and 2.75 mmol/L potassium persulfate solution. ABTS mother liquor was prepared by mixing 10 mL of ABTS solution with 10 mL of potassium persulfate solution and then placed in a dark environment for 12–16 h at room temperature. The OD 734 nm of 7 mmol/L ABTS mother solution was adjusted to 0.71 using PBS to obtain ABTS free radical solution; 100 µL of LAB solution was added to 3 mL of ABTS free radical solution and protected from light for 6 min. The absorbance was measured at OD 734 nm (Ab0). The absorbance was measured in the control group by replacing the sample solution with PBS (Ab1) [24]. The clearance was calculated as follows:

(Ab0 − Ab1)/Ab0 × 100.

#### 2.8.3. Determination of the Scavenging Capacity of Superoxide Anion Radicals

Take 0.1~0.5 mL of LAB solution (add distilled water to make up to 0.5 mL) in a 10 mL stoppered glass test tube, add 4.5 mL of 50 mmol/L Tris-hydrochloric acid buffer solution, and water bath at 25 °C for 15 min, add 0.4 mL of 10 mmol/L o-benzenetriol, shake well, and leave it for 5 min in a water bath at 25 °C, and add 0.1 mL of 8 mol/L hydrochloric acid. The reaction was terminated, and the absorbance (Ac0) was measured immediately at OD 325 nm [25]. Ac0 was obtained by replacing o-triphenol with an equal volume of distilled water, Ac2 was obtained by substituting the extract with an equal volume of distilled water, and the clearance was calculated using the following formula:

(1 − (Ac0 − Ac2)/Ac1) × 100.

### 2.9. Safety Evaluation of Lactic Acid Bacteria

#### 2.9.1. Amino Acid Decarboxylase Activity

MRS liquid medium containing 1% lysine, arginine, histidine, and tyrosine and 0.6 g/L bromocresol violet was prepared, filtered, and sterilized, and 1% (*v*/*v*) of the activated bacterial solution was inoculated into the four MRS liquid mediums, and the blank liquid medium was used as the control group, which was incubated at 37 °C for 4 d [26]. The color of the medium becoming yellow indicates that the result is negative, while a change to purple was considered positive.

#### 2.9.2. Hemolysis

To assess the hemolytic activity, selected isolates were incubated in a blood agar medium supplemented with 5% sheep’s blood (*v*/*v*) for 24 h at 37 °C [27]. The appearance of green hemolytic circle around the colonies was considered as α-hemolysis, the appearance of a transparent hemolytic circle around the colonies was considered as β-hemolysis, and the absence of a hemolytic circle was considered as γ-hemolysis. The results were compared with the hemolytic effect of *Staphylococcus aureus*.

#### 2.9.3. Antibiotic Resistance

The drug-sensitive agar diffusion method was used for the drug-sensitivity test, and the overnight cultured bacterial solution was inoculated into MRS solid medium at 0.2% (*v*/*v*). After the plate solidified, antibiotic paper (6 mm in diameter) was placed uniformly on the plate under aseptic conditions with sterile tweezers. The diameter of the circle of inhibition was measured [28] after incubation for 24 h at 37 °C. The drug-sensitive papers used were tetracycline (30 μg/tablet), kanamycin (30 μg/tablet), ampicillin (10 μg/tablet), gentamicin (10 ± 2.5 μg/tablet), erythromycin (15 μg/tablet), chloramphenicol (30 μg/tablet), clindamycin (20 μg/tablet), vancomycin (30 μg/tablet), and streptomycin (30 μg/tablet).

#### 2.9.4. Bacteriostatic Properties

*Staphylococcus aureus* (ATCC25923), *Escherichia coli* (ATCC25922), and *Bacillus cereus* (CI-CC21290) were used as indicator organisms and cultured using lysogeny broth (LB) medium. The inhibitory activity of the isolates against *Staphylococcus aureus*, *Escherichia coli*, and *Bacillus cereus* was evaluated using an Oxford cup [29]. Briefly, Petri dishes (10 cm) with 5 mL of LB agar and sterilized Oxford cups covered with 10 mL of soft LB agar were pipetted with 100 μL of pathogenic bacterial fluids (10^6^~10^7^ CFU/mL) onto nutrient agar plates at 45 °C. After solidification of the medium, the Oxford cup was removed, and 150 µL of activated LAB was added to the wall and left to stand at 4 °C for 2 h. The plates were incubated at 37 °C for 24 h. The antipathogenic activity of the bacteria was determined by measuring the diameter of the zone of inhibition around the LAB isolation site.

### 2.10. Data Processing

We repeated the test three times and processed the results using the statistical software Origin 2018, Excel 2019, and SPSS 23. One-way ANOVA was used to test the significance of differences. Mean values were verified post hoc using Duncan’s multiple range test.

## 3. Results and Discussion

### 3.1. Results of Isolation and Purification of Strains

**Figure 1:**
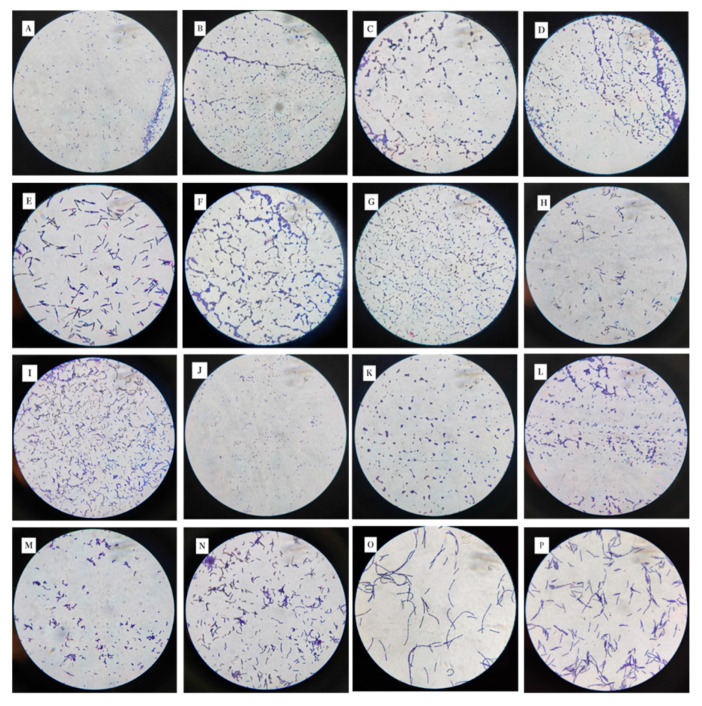
The 16 strains isolated from the samples were magnified 100 times under optical microscopes, and the serial numbers (**A**–**P**) correspond to the colony morphology under optical microscopes of strain numbers 1–16, respectively.

A total of 16 suspected LAB colonies were isolated and screened out with obvious calcium solubilizing rings from the Boza samples, and the colonies were mainly transparent, translucent, white or creamy white in color, with rounded, smooth, and slightly elevated surfaces. The results of microscopic examination were mostly short or long rods. The 16 strains of suspected LAB were Gram stain positive and catalase negative; they can ferment various sugars, including fructose, glucose, lactose, and sucrose, but the growth performance in arabinose and xylose is slightly inferior, while the growth in trehalose shows certain differences. This difference in growth in different sugars suggests that these strains may have diversity at the genus or species level. In order to further clarify its taxonomic status, it was necessary to combine molecular identification techniques. At the same time, based on safety (such as hemolysis and drug resistance) and functional (such as acid bile salt resistance and antibacterial activity) indicators, candidate strains suitable for food industry applications were screened. Based on the above results, the 16 strains conformed to the physiological and biochemical characteristics of LAB. The colony morphology of LAB with Gram staining is shown in Figure 1.

**Figure 2:**
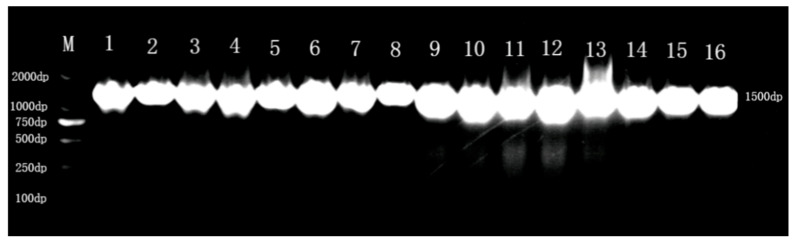
DNA gel electrophoresis imaging of the 16 strains.In the figure, M represents the maker, the value on the left represents the corresponding fragment position of the maker, and the values 1–16 represent the strain abbreviation number of strains 1–16.

DNA extraction and PCR amplification were carried out on the 16 strains of LAB preliminarily identified, and the results of PCR amplification are shown in Figure 2. The 16 strains of LAB 16S rDNA were amplified and analyzed by 1% agarose gel electrophoresis, which revealed that a single bright band appeared near 1500 bp for the 16 strains of LAB, and the results of the gene sequences obtained by sequencing of the amplified products were compared with the known sequences on the NCBI website.

**Figure 3:**
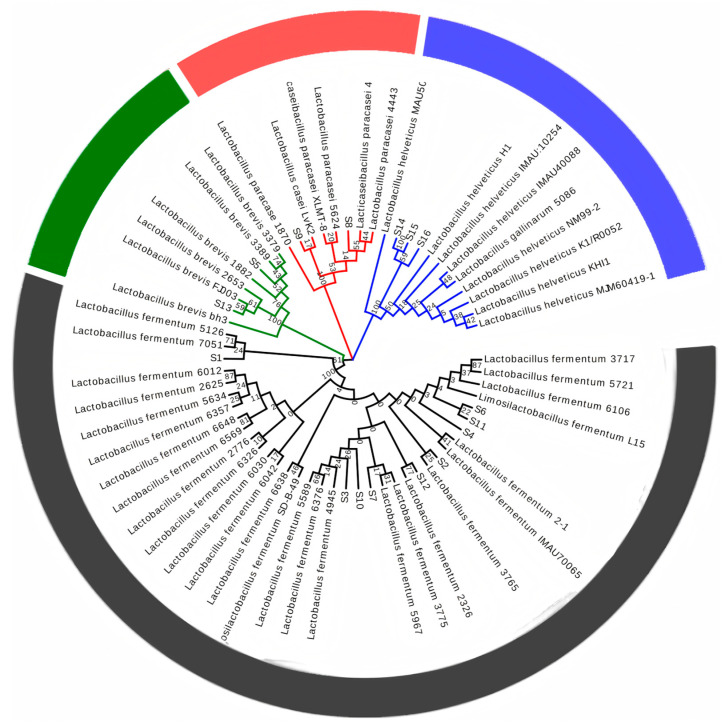
Using MEGA11 software, the molecular phylogenetic analysis of the 16S rDNA sequence of the isolated 16 strains was carried out by the maximum likelihood method, and the branch tips were colored according to the genus classification. Different colors represent the classification of strains.

As shown in Figure 3, a total of four species of the 16 strains were identified, including 9 strains of *Limosilactobacillus fermentum* (56.25% of the total), 2 strains of *Levilactobacillus brevis* (12.5% of the total), 2 strains of *Lacticaseibacillus paracasei* (12.5% of the total), and 3 strains of *Lactobacillus helveticus* (18.75% of the total). These results indicated that *Limosilactobacillus fermentum* was the first dominant strain in the traditional fermented Boza. Similar to other fermented products, *Limosilactobacillus fermentum* was also isolated and identified as the dominant strain in teff injera dough fermen [30], New Zealand rice sourdough [31], and soybean paste [32]. According to the information we have, *Levilactobacillus brevis*, *Limosilactobacillus fermentum*, *Lactobacillus helveticu*, and *Lacticaseibacillus paracasei* belong to the category of probiotics and demonstrate high safety. Through drug resistance tests, hemolytic tests, and acute oral toxicity tests [33] (such as applications in mouse models), it has been confirmed that these strains do not produce toxins, do not have hemolytic activity, and do not have pathogenic risk; in particular, *Lactobacillus helveticus*, due to its long history of safe applications, has been awarded the GRAS (recognized safety) certification [34]. In addition, *Levilactobacillus brevis* can widely inhibit a variety of pathogenic bacteria, including *Staphylococcus aureus* and *Salmonella*, and has good nitrite reduction ability [35]; *Limosilactobacillus fermentum* showed good anti-biofilm activity against intestinal pathogens such as *Escherichia coli* [36]. In addition, *Lactobacillus helveticus*, which accounted for 18.75% of the total, was the second dominant strain identified in this study, and this bacterium has been less reported in Boza.

### 3.2. Fermentation Characterization of Lactic Acid Bacteria

**Figure 4:**
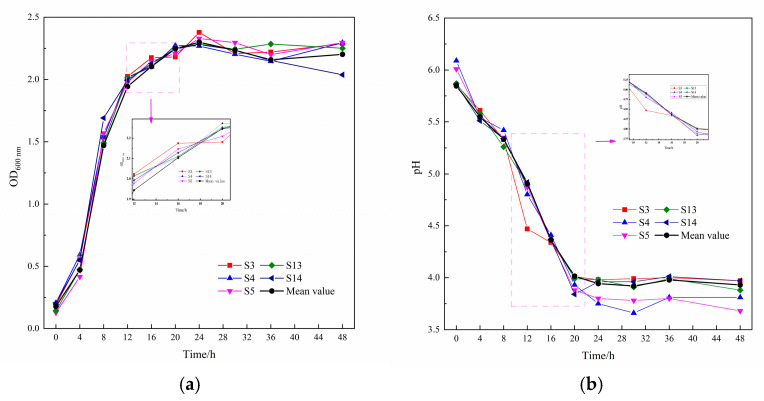
The growth rate and acid production ability of five lactic acid bacteria: (**a**) growth curve and (**b**) pH dynamic of the culture medium.

In an acidic environment, a rapid decrease in pH can significantly inhibit the growth and multiplication of acid-sensitive microorganisms, thus ensuring product quality and safety during the fermentation process. In addition, strains with higher growth rates can quickly take advantage and ferment the samples. Therefore, when screening LAB, priority should be given to those strains that can produce acids quickly, reduce pH effectively, and have excellent growth rates. Figure 4 shows five strains of LAB with efficient acid production and growth rates. Acid production and growth rate curves of the sixteen strains of LAB were fitted to obtain a mean curve, and the fermentation rates of the five LAB strains were better than the mean value of all strains. The five LAB strains included two strains of *Limosilactobacillus fermentum* (S3, S4), two strains of *Levilactobacillus brevis* (S5, S13), and one strain of *Lactobacillus helveticus* (S14). These strains produced acid rapidly during the first 24 h, and the pH dropped below 4.5 within 20 h. Especially for strains S4 and S5, the pH dropped below 3.8 within 24 h. After 24 h, the rate of acid production of the five strains gradually leveled off, stabilizing the pH of the MRS liquid medium between 3.65 and 3.9. The growth rate of strain S4 was faster, and strain S5 had the shortest delay period, entered the logarithmic phase at about 4 h, and had a high bacterial population at 30 h. Strain S3 had a longer delay period and a short logarithmic growth period but had a high bacterial population. The chart data showed that after 30 h of culture, the number of viable bacteria in S5 and S15 reached the peak, showing the most significant growth activity. In summary, *Levilactobacillus brevis* S5 has the potential for rapid fermentation of food due to its brief adaptation period and long-lasting logarithmic growth period; *Limosilactobacillus fermentum* S4 can effectively inhibit foodborne pathogens and shorten the fermentation cycle due to its rapid acid production capacity. However, it must be pointed out that the excessive acid production of a single strain may have a negative impact on the texture of the product. Therefore, it is recommended to use mild strains for compounding to balance the fermentation process and verify its multifunctional synergistic effect.

**Figure 5:**
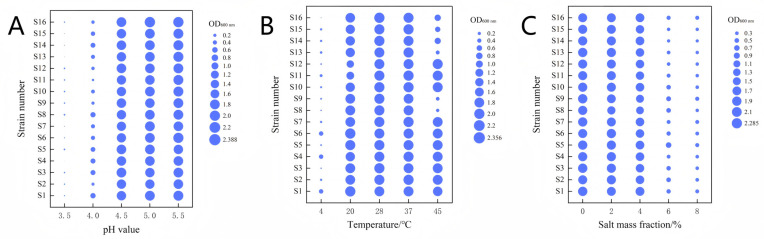
Evaluation of the acid resistance, temperature resistance, and salt tolerance in the 16 strains. (**A**) The growth of the 16 strains under different acidic conditions (pH 3.5, 4.0, 4.5, 5.0, and 5.5); (**B**) the growth of the 16 strains at different temperatures (4 °C, 20 °C, 28 °C, 37 °C, and 45 °C); (**C**) the growth of the 16 strains at different NaCl concentrations (0%, 2%, 4%, 6%, and 8%). The size of the bubbles in the figure represents the OD 600 nm absorbance value of each strain after 24 h of culture under different growth environment conditions.

During the fermentation process of LAB, the content of organic acids gradually accumulates, whereas the pH value of the fermentation environment continues to decrease. Therefore, the screened LAB must have excellent acid tolerance to ensure that they can effectively exert their fermentation properties under acidic conditions. The optical density values (OD 600 nm) of the 16 LAB strains shown in Figure 5A decreased continuously with a decrease in medium pH. Among them, S2, S9, S10, and S11 were the least acid-tolerant, and the density values of the other strains in different pH media were as follows: The density ratios ranged from 75.20% to 86.92% at pH 5, and the density ratios of six strains were still greater than 20% at pH 4, including three strains of *Limosilactobacillus fermentum* (S1, S3, and S4), one strain of *Levilactobacillus brevis* (S5), one strain of *Lacticaseibacillus paracasei* (S8), and one strain of *Lactobacillus helveticus* (S14); all strains had density ratios less than 5% at pH values below 4. These results showed that the 16 strains still had good tolerance in low-pH environments, among which S1, S3, S4, S5, S8, S14, and S15 had the best acid tolerance.

Fermentation temperature, as a key factor affecting the fermentation of LAB, is decisive for the flavor and texture of fermentation products. The ambient temperature greatly influences the growth and metabolism of LAB, causing their growth and reproduction to vary under different temperatures. The optimal growth temperature of LAB is usually 25–37 °C [37]. However, some LAB can still show strong growth ability and adaptability under low-temperature environments. As can be seen from Figure 5B, the optical density values of all strains showed a decreasing trend with decreasing temperature, and when the temperature dropped to 4 °C, the optical density values of all strains decreased to less than 0.50, among which strains S1 and S4 showed relatively high survival rates with strain density ratios > 5%. Under the condition of 37 °C, which is the standard temperature in the traditional fermentation process, all 16 strains of LAB were able to achieve satisfactory growth with an absorbance value of 2.0 or above and a strain density ratio of >90%; when the temperature was 45 °C, the growth of some strains was inhibited, but *Limosilactobacillus fermentum* still showed relatively strong viability. From these results, it can be seen that the temperature has an important influence on the growth of LAB, and the growth of the strains was inhibited under the low-temperature environment.

The growth and reproduction of microorganisms require ideal osmotic pressure conditions, and osmotic pressure is too large to inhibit the growth and reproduction of microorganisms or even cause dehydration death [38]. Figure 5C displays the salt tolerance of the 16 strains of LAB. It can be seen that the OD 600 nm values of all strains decreased with increasing salt content, and when the salt mass fraction reached 8%, the OD 600 nm values of all strains were lower than 0.45, which was consistent with the results of Fan et al. [39]. When the salt mass fraction was 4%, all 16 strains of LAB were able to grow well, with absorbance values above 1.5, and the density ratio of the strains was >65.22%; when the salt mass fraction was 6%, strains S2, S5, S8, S9, and S10 still showed relatively strong survival ability, and the density ratio of the strains was >23.91%. From these results, it can be seen that salt concentration has an important effect on the growth of LAB, and the growth of strains was significantly inhibited under high salt environments.

### 3.3. Simulated Human Gastrointestinal Environmental Tolerance Analysis

**Figure 6:**
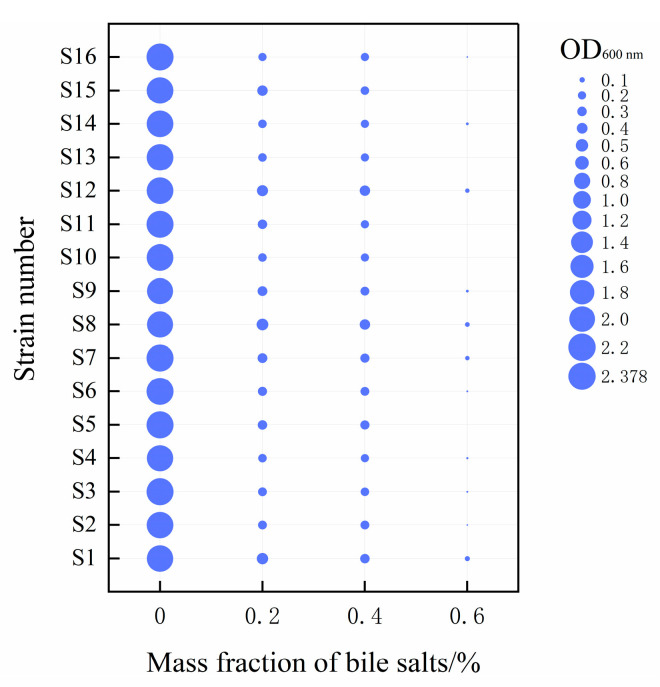
The growth of the 16 strains of bacteria under different bile salt concentrations was evaluated. The size of the bubbles in the figure represents the OD 600 nm absorbance value of each strain after 24 h of culture under different bile salt concentrations.

Bile salt tolerance is an important factor in the selection of probiotic strain [40]. The mass fraction of bile salts in the human small intestinal tract is about 0.3%, so if the probiotic bacteria are allowed to play a beneficial role in the human body, the selected strains should have a specific tolerance to bile salts in order to maintain enough live bacteria to fulfill their beneficial role. The results of bile salt resistance of the strains are shown in Figure 6. In the study, the 16 strains of LAB were able to grow at 0.2% and 0.4% bile salt concentration, but their growth ability was inhibited with increasing bile salt concentration, and the growth was poor at 0.6% bile salt concentration. Among them, two strains of *Limosilactobacillus fermentum* (S1 and S12) and one strain of *Lacticaseibacillus paracasei* (S8) showed adequate bile salt tolerance. When the bile salt mass fraction reached 0.2%, the OD 600 nm values of the three strains of LAB exceeded 0.45, and the strain density ratio exceeded 17%. When the bile salt content was further increased to 0.6%, four strains (S1, S7, S8, and S12) still showed survival ability, and the strain density ratio was about 2%. In addition, it was found that most *lactobacilli* could grow at bile salt concentrations ranging from 0.2% to 0.5%, and a few strains were able to tolerate higher bile salt concentrations [40,41], which may be related to the fatty acid composition of LAB and the bile salt hydrolytic enzymes secreted by the LAB themselves [42].

The low pH in the gastric fluid of the host and the bile salts in the intestinal fluid are important barriers that affect the activity of probiotics after they have passed through the digestive tract, while the survival of the strains is the basis for determining their probiotic properties [43]. Human gastric fluid typically has a pH of 2.5, while solid foods stay in the stomach and undergo digestion for approximately 3 h. This process kills most microorganisms [44]. Pepsin and trypsin in gastric and intestinal fluids are also coercive to microorganisms. Therefore, digestive tract tolerance is an important indicator for evaluating whether microorganisms have probiotic potential. As shown in Table 1, all strains remained highly active after 3 h of incubation in simulated gastric fluid. Except for strains S13 and S5, the survival rate of all strains was higher than 50%, indicating that the isolated and obtained lactobacilli had forceful gastric environment tolerance ability. After 6 h of incubation with simulated intestinal fluid, all strains except strain S7 were still highly active, with the highest viable counts being *Lacticaseibacillus paracasei* S9 (36.4%) and *Limosilactobacillus fermentum* S6 (28.9%). Strain S9 (*Lacticaseibacillus paracasei*) showed strong tolerance in simulated gastric fluid (survival rate more than 50%) and intestinal fluid (survival rate more than 25%), which proved its potential probiotic properties through the digestive tract barrier. This feature may be attributed to the strong bile salt hydrolase activity of the S9 strain. Similar to this result, Leeuwendaal, et al. [45] isolated specific strains from Cheddar cheese and evaluated their probiotic properties, such as bile salt hydrolase activity, ability to adhere to intestinal epithelial cells, and inhibition of intestinal pathogen binding. The results showed that *Lacticaseibacillus paracasei* and *Lactobacillus rhamnosus* showed significant probiotic potential. These strains show acceptable survival rates in simulated digestive environments and may have positive effects on health.

### 3.4. Analysis of In Vitro Characterization and Evaluation of Probiotic Properties

**Figure 7:**
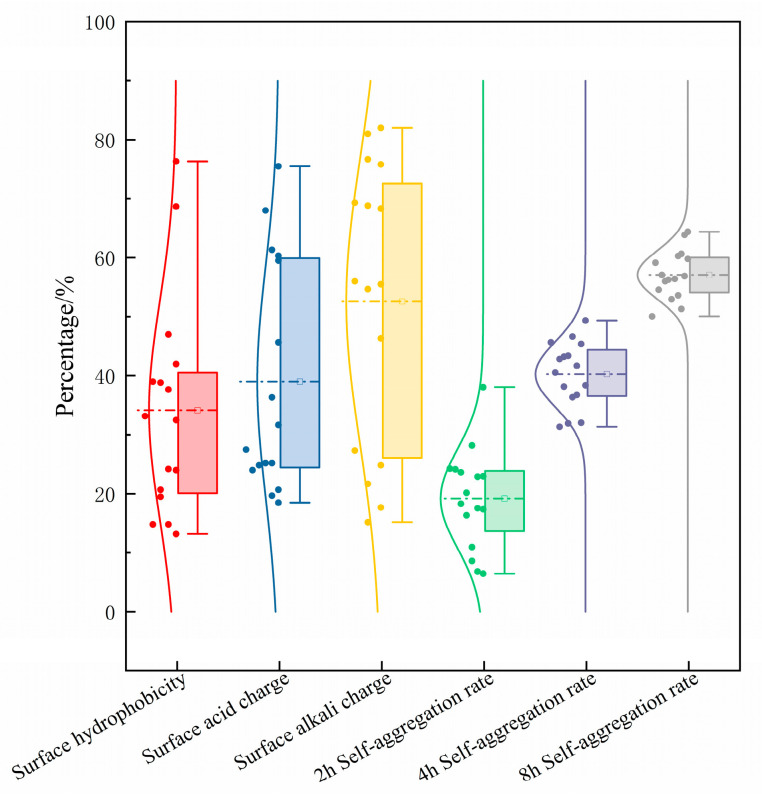
Box plots of surface hydrophobicity, surface acidic charge, surface alkaline charge, and self-aggregation ability of the 16 strains of lactic acid bacteria at 2 h, 4 h, and 8 h. The dotted lines in the figure represent the average of the corresponding values.

Probiotics need the ability to adhere to intestinal mucus in order to colonize and multiply in the gut [46]. In this study, we indirectly assessed the adhesion ability of the 16 strains of LAB by determining their hydrophobicity, surface acid–base charge, and self-aggregation ability. Hydrophobicity refers to the ability of a bacterium to detach from the aqueous phase and adhere to other organic phases. The nonspecific adhesion ability of bacteria closely correlates with their hydrophobicity [47]. The more hydrophobic a bacterium is, the more likely it is to adhere to the surface of the gastrointestinal tract of the human body and thus to exert a probiotic effect [48]. We categorized the hydrophobicity of the strains into three levels: those less than 40% as hydrophobic, those between 40% and 60% as moderately hydrophobic, and strains with hydrophobicity more than 60% are categorized as highly hydrophobic. As shown in Figure 7, the surface hydrophobicity of the 16 strains of LAB ranged from 13.17% to 76.33%, with the average value reaching 34.15%. Strains S1 and S2 were highly hydrophobic, with 76.33% and 68.67%, respectively. Strains S4 and S16 were moderately hydrophobic, and the other strains had low hydrophobicity; strains S8 and S10 were hydrophobic by only 13.17% and 14.83%, respectively, indicating that strains S8 and S10 had poor intestinal colonization ability, which might be related to the strain specificity.

Hydrophobicity and acid–base charge of the bacterial surface correlate with nonspecific adhesion between strain and host. When the bacterial surface has a lower acid charge and a higher alkaline charge, it does not promote biofilm formation and is more conducive to adhesion to epithelial cells [21]. Therefore, we examined the distribution of acid and alkaline charges on the surface of LAB. Strain S3 and strain S5 had relatively low surface acid charges of 18.50% and 19.67%, respectively, followed by strain S1 with a surface acid charge of 20.67%. Strain S3 and strain S13 had relatively high surface alkali charges of 82.00% and 81.00%, respectively. In conclusion, strain S3 has a low surface acid charge and a high surface base charge and is presumed to have favorable intestinal colonization ability.

The ability to adhere to the intestinal epithelium is a key criterion for the selection of probiotic strains. Assessed by microbial adhesion to hydrocarbons, strains adhering to cell monolayers typically showed strong correlations with self-aggregation and hydrophobic properties [15]. The self-aggregation capacity of all strains increased with incubation time, and all reached the highest self-aggregation value after 8 h of incubation. In general, strains with high self-aggregation capacity are in an advantageous position when competing for cell–host binding sites, which facilitates their colonization of the intestine and effectively prevents pathogen–host binding, thus preventing adherence to the gastrointestinal tract of pathogenic bacteria [47]. The self-aggregation ability of strains S1, S4, S6, and S15 was consistently higher than the average value; these four strains coalesced rapidly within the first 2 h, exceeded 43% at 4 h, and showed a slight increase in self-aggregation rate at 8 h. We hypothesized that strains S1, S4, S6, and S15 might have a high ability to adhere to intestinal epithelial cells.

### 3.5. Study on Antioxidant Properties of Lactic Acid Bacteria

**Figure 8:**
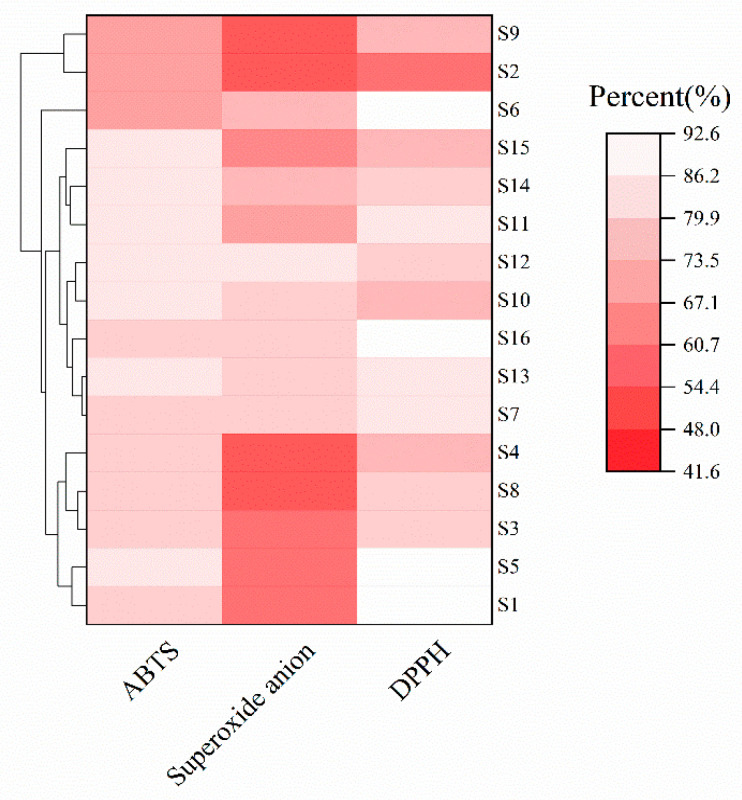
Heatmap of ABTS, superoxide anion, and DPPH phenotypes of the 16 strains of lactic acid bacteria.

Free radicals such as DPPH radicals, ABTS radicals, and superoxide anion radicals in the organism are highly oxidative and produce oxidative stress that can lead to damage to DNA, proteins, small cell molecules, etc., which in turn causes oxidative damage to the organism [49]. Therefore, it is critical to evaluate the antioxidant activity of strains. Some studies have reported that the accuracy in determining the antioxidant activity of strains only based on the criterion that scavenging rate of a single free radical is not high. In order to improve the validity of the results, the scavenging rate of a variety of free radicals should be determined comprehensively [50]. In this study, the in vitro antioxidant activity of the 16 strains of LAB was analyzed by determining the DPPH radical scavenging energy, ABTS radical scavenging capacity, and superoxide anion radical scavenging capacity (Figure 8). Overall, all 16 strains of LAB had some in vitro antioxidant capacity. The DPPH radical scavenging ability of the fermentation supernatant of the 16 strains of LAB ranged from 51.24% to 92.45%, the scavenging ability of ABTS radicals ranged from 61.21% to 82.89%, and the scavenging rate of superoxide anion radicals ranged from 41.66% to 82.33%. Among the 16 strains, strain S1 had the highest scavenging capacity for DPPH radicals, strains S6 and S16 also showed strong scavenging activity for DPPH radicals, and strain S2 had the lowest scavenging capacity for DPPH radicals. It is noteworthy that despite the high DPPH radical scavenging capacity of strains S1 and S6, strains S1 and S16 showed a weaker scavenging capacity for ABTS (75.49% and 62.74%, respectively). Strains S6, S7, S10, and S12 of the *Limosilactobacillus fermentum* group had relatively outstanding superoxide anion radical scavenging ability, whereas S2 and S4 of *Limosilactobacillus fermentum* had poor superoxide anion radical scavenging ability, suggesting that the radical scavenging activities of strains also differed to some extent among strains of the same genus. In addition, the ABTS radical scavenging rate and DPPH radical scavenging rate of *Levilactobacillus brevis* group S5 and S13 were higher than those of *Lacticaseibacillus paracasei* group S8 and S9 and most of the *Limosilactobacillus fermentum* group, suggesting that *Levilactobacillus brevis* group S5 and S13 have a better free radical scavenging rate capacity. Strains with good antioxidant activity can be applied as potential antioxidant strains for functional food development, which can increase the total phenolic content and antioxidant capacity of fermentation products and help to prevent and control oxidative stress-related diseases, thus increasing their potential health-promoting effects [51].

### 3.6. Evaluation of Potential Safety Risks of Lactic Acid Bacteria

Microorganisms with amino acid decarboxylase activity act on amino acids to decarboxylate them, which promotes the formation of biogenic amines in food, leading to the accumulation of biogenic amines and thus posing a hazard to human health [52]. Therefore, screening strains without the ability to decarboxylate amino acids as fermenters can effectively control the biogenic amine content and improve the safety of fermentation. All strains were amino acid decarboxylase negative and can be used as fermenter candidates.

Safety evaluation is a prerequisite for whether a bacterium can be used as a probiotic, and the detection of hemolytic activity of the strains is an important indicator for the safety evaluation of probiotics [53]. The 16 strains of LAB were cultured for 48 h on Colombian blood plates without hemolytic circles appearing around the colonies. The results showed that the 16 strains with probiotic potential isolated from Boza and their metabolites had no damage to red blood cells.

According to the study reports, two types of antibiotics are usually used as indicators for the evaluation of probiotic strains: cell wall synthesis inhibitors, including ampicillin and vancomycin, and protein synthesis inhibitors, including chloramphenicol, gentamicin, clindamycin, erythromycin, streptomycin, kanamycin, and tetracycline [54]. Combining the common antibiotics in most studies, tetracycline (30 μg/tablet), kanamycin (30 μg/tablet), ampicillin (10 μg/tablet), gentamicin (10 ± 2.5 μg/tablet), erythromycin (15 μg/tablet), chloramphenicol (30 μg/tablet), clindamycin (20 μg/tablet), vancomycin (30 μg/tablet), and streptomycin (30 μg/tablet) were selected for resistance testing. The susceptibility classes were classified into resistant (R), intermediate (I), and sensitive (S) according to the relevant research criteria [55], and the results are shown in Table 2. Studies have found that most of the strains were sensitive to tetracycline, ampicillin, chloramphenicol, erythromycin, and clindamycin, and resistant to kanamycin, gentamicin, vancomycin, and streptomycin, which may be due to the fact that LAB have an intrinsic resistance [28]. Specifically, eight strains of *Limosilactobacillus fermentum* were sensitive to erythromycin and clindamycin; two strains of *Lactobacillus helveticus* were intermediary to clindamycin, and one strain was intermediary to ampicillin, which accounted for about 67% and 34% of the total number of strains, respectively; all the *Lacticaseibacillus paracasei* strains were sensitive to tetracycline, ampicillin, chloramphenicol, erythromycin, and clindamycin; one strain of *Levilactobacillus brevis* was intermediary to tetracycline and ampicillin; all LAB were susceptible to chloramphenicol; *Limosilactobacillus fermentum* S6, *Levilactobacillus brevis* S13, and *Lactobacillus helveticus* S14 had better antibiotic resistance. Similar to this result, Hossein et al. [27] isolated and screened 30 strains of LAB from Duimaj, a traditional Iranian recreational food, and they were generally resistant to kanamycin and vancomycin, streptomycin, and kanamycin. Parlindungan et al. [28] isolated and screened 22 strains of LAB from fermented meat products, and these were generally found to be resistant to vancomycin, gentamicin, and streptomycin. Zeng et al. [29] reported similar resistance results for 18 strains of LAB isolated from pineapple fermentation residue.

The presence of foodborne pathogenic microorganisms in food accelerates food spoilage and poses health risks. At present, many studies have found that the metabolites extracted from probiotics and their fermentation products have the inhibiting effect on foodborne pathogenic microorganisms and have the potential to be applied in food safety and quality control [56]. Table 2 evaluates the bacteriostatic effect of the 16 strains of LAB against 3 standard strains (*Staphylococcus aureus*, *Escherichia coli*, and *Bacillus cereus*). As shown in Table 2, all 16 tested strains were able to inhibit *Staphylococcus aureus*, while the diameter of the circle of inhibition against *Bacillus cereus* and *Escherichia coli* was not significant. The antimicrobial capacity of LAB is related to the antimicrobial substances produced by the strains, such as organic acids, hydrogen peroxide, and bacterial peptides [57]. Yang et al. [58] noted that *Lactobacillus plantarum* LR-14 isolated from Sichuan kimchi caused both cellular damage and effective inhibition and removal of bioepithelial membrane formation against *Staphylococcus aureus*. Petkova et al. [59] showed that eleven strains of LAB screened from Bulgarian sourdough had similar inhibitory effects against *Staphylococcus aureus*.

### 3.7. PCA-Based Phenotypic Synthesis of Strains

**Figure 9:**
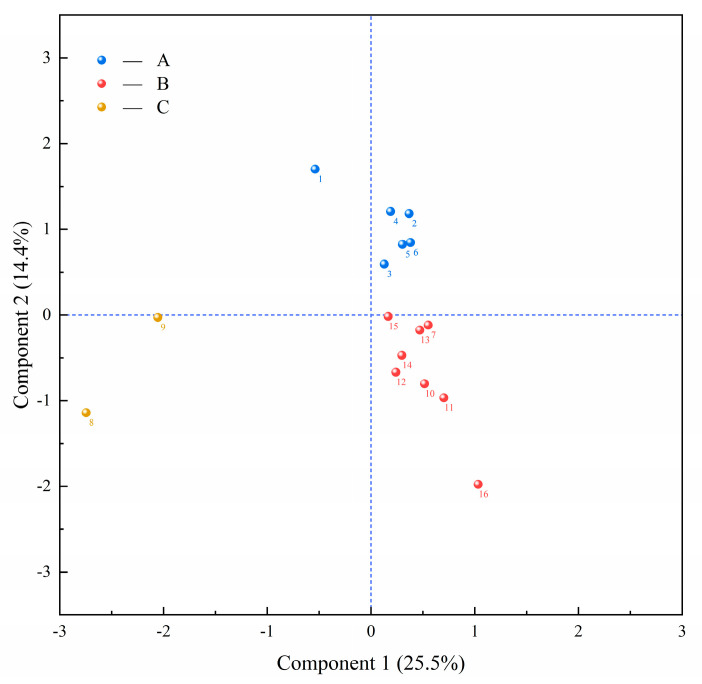
The principal component analysis (PCA) method was used to comprehensively evaluate the strain based on its fermentation performance and probiotic performance. The blue, yellow, and red dots in the figure represent three different strain groups divided according to similarity, and each number corresponds to the specific strain.

The comprehensive performance of the isolated strains was evaluated based on the phenotypic comprehensive analysis of PCA, and PCA plots were drawn using fermentation performance and probiotic performance as indexes; the results are shown in Figure 9. The PCA plots explained 39.9% of the total variance, indicating that there was a significant difference in phenotypes of the tested strains. The plot is divided into three groups according to the phenotypic similarity, the strains in group A with better overall performance. Strain S1 in group A had higher antibiotic sensitivity and weaker adaptation to the environment. Strain S2 had poor DPPH radical clearing ability and acid tolerance, and was unable to quickly adapt to the low-acid fermentation environment and play a probiotic role; strain S3 showed different disadvantages in bile salt resistance and gastrointestinal tolerance compared with the other strains in the same group; strains S4 and S5 had better performance in the combination of fermentation properties such as growth characteristics, acid production, and acid tolerance. Additionally, strain S6 had good inhibitory effect and strong gastrointestinal fluid tolerance ability. Strains S5 and S6 also had good ability in DPPH radical scavenging rate, superoxide anion scavenging ability, gastrointestinal adhesion ability, and other probiotic properties. Principal component analysis (PCA) revealed the complementarity of S4, S5, and S6 in terms of fermentation performance and probiotic characteristics: S4 showed rapid acid production ability, S5 showed significant growth kinetic advantages, and S6 relied on its antibacterial activity and tolerance to become a probiotic carrier. Although S5 is sensitive to simulated gastric juice, its very short lag period (4 h) can mitigate its survival disadvantage; the synergistic effect of the antibacterial breadth of S6 and the antioxidant capacity of S5 provide a theoretical basis for the formulation. Therefore, strains S4, S5, and S6 can be considered potential starter candidates.

Therefore, strains S4, S5, and S6 can be used as potential starter candidate strains.

## 4. Conclusions

In this study, 16 strains conforming to the physiological and biochemical characteristics of LAB were isolated and screened from Boza. Through the 16S rDNA sequence comparison analysis, the results showed that *Limosilactobacillus fermentum* was the dominant LAB in Boza, accounting for 56.25% of the isolated strains. The fermentation and probiotic properties of the 16 strains of LAB were tested, and finally 3 strains, *Limosilactobacillus fermentum* S4 and S6 and *Levilactobacillus brevis* S5, which revealed high acid production and growth rates, acid tolerance, higher gastrointestinal fluid tolerance, and free radical scavenging ability, were selected by principal component analysis. The phenotypical excellence of the strains S4, S5, and S6 showed their potential as candidate strains for excellent starter culture. Future studies can further explore the probiotic properties of the strains based on cells and live animals and carry out in-depth studies on their specific probiotic functions. The present study may provide a theoretical basis for the discovery of grain-derived LAB and the enrichment of the strain resource base.

## Figures and Tables

**Table 1:** The tolerance of the 16 strains of LAB to simulated gastrointestinal fluid.

Strain Number	Gastric Survival	Intestinal Survival
	(VI%)	(VI%)
S1	52.0 ± 4.2 ^gh^	28.6 ± 0.4 ^bc^
S2	66.4 ± 2.0 ^ef^	24.6 ± 0.7 ^bcde^
S3	58.7 ± 1.2 ^fg^	20.5 ± 1.0 ^de^
S4	97.6 ± 7.9 ^a^	24.4 ± 0.4 ^bcde^
S5	45.9 ± 5.6 ^hi^	21.4 ± 3.5 ^de^
S6	64.7 ± 5.3 ^f^	28.9 ± 6.8 ^b^
S7	69.0 ± 1.2 ^ef^	19.2 ± 3.8 ^e^
S8	96.2 ± 11.8 ^ab^	24.8 ± 4.4 ^bcde^
S9	85.6 ± 2.4 ^bcd^	36.4 ± 3.5 ^a^
S10	76.8 ± 6.4 ^de^	21.8 ± 3.4 ^cde^
S11	84.4 ± 6.1 ^cd^	20.4 ± 1.2 ^de^
S12	66.8 ± 3.0 ^ef^	19.6 ± 0.4 ^e^
S13	40.4 ± 2.7 ^i^	25.9 ± 5.5 ^bcde^
S14	85.2 ± 6.9 ^bcd^	20.8 ± 4.0 ^de^
S15	86.5 ± 1.1 ^abcd^	28.8 ± 4.0 ^b^
S16	91.9 ± 8.6 ^abc^	26.8 ± 1.6 ^bcd^

Note: S1–S16 represent different strains, and different letters represent significant differences between the two groups.

**Table 2:** Determination of beneficial activity of the 16 strains of LAB.

Strain Number	S1	S2	S3	S4	S5	S6	S7	S8	S9	S10	S11	S12	S13	S14	S15	S16
Tetracycline (TET)	S	S	S	S	S	S	S	S	S	S	S	S	I	S	S	S
Kanamycin (KAN)	I	R	R	R	R	R	R	R	R	R	R	R	R	I	I	R
Ampicillin (AMP)	S	S	S	S	S	S	S	S	S	S	S	S	I	I	S	S
Gentamycin (GEN)	I	I	I	I	I	R	I	I	I	I	I	I	R	R	R	I
Erythromycin (E)	S	S	S	S	S	R	S	S	S	S	S	S	S	S	S	S
Chloramphenicol (C)	S	S	S	S	S	S	S	S	S	S	S	S	S	S	S	S
Clindamycin (CC)	S	S	S	S	S	I	S	S	S	S	S	S	S	I	I	S
Vancomycin (VAN)	S	S	I	S	S	I	S	S	S	S	S	S	R	I	R	S
Streptomycin (S)	I	R	R	R	R	R	R	R	R	R	R	R	R	R	R	R
*Staphylococcus aureus*	+	+	+	+	+	+	+	+	+	+	+	+	+	+	+	+
*Colibacillus*	−	−	−	−	−	−	−	−	−	−	−	−	−	−	−	−
*Bacillus cereous*	−	−	−	−	−	−	−	−	−	−	−	−	−	−	−	−

Note: R means resistance, I means intermediary, S means sensitive, + means antibacterial, − means the inhibition zone is not obvious.

## Data Availability

Data sharing can be provided upon request, and the data required for scientific research in accordance with the laws of the People’s Republic of China can be obtained by contacting the corresponding authors. Data will be provided upon approval.
